# Caregiver perspectives on the continued impact of the COVID-19 pandemic on children with intellectual/developmental disabilities

**DOI:** 10.3389/fped.2023.1196275

**Published:** 2023-08-07

**Authors:** R. A. Northrup, E. Jones, V. Singh, C. Holingue, M. Meck, C. A. Gurnett, M. van Stone, L. G. Kalb

**Affiliations:** ^1^ Department of Neuropsychology, Kennedy Krieger Institute, Baltimore, MD, United States; ^2^ Information Systems Department, Kennedy Krieger Institute, Baltimore, MD, United States; ^3^ Center for Autism and Related Disorders, Kennedy Krieger Institute, Baltimore, MD, United States; ^4^ Department of Mental Health, Johns Hopkins Bloomberg School of Public Health, Baltimore, MD, United States; ^5^ Maryland Center for Developmental Disabilities, Kennedy Krieger Institute, Baltimore, MD, United States; ^6^ Department of Neurology, Washington University School of Medicine in St. Louis, St. Louis, MO, United States

**Keywords:** mental health, pandemic, COVID-19, intellectual disability, healthcare, services, caregivers, developmental disability

## Abstract

The COVID-19 pandemic has significantly impacted caregivers, especially those raising a child with an intellectual/developmental disability (IDD). While research has shown substantial disruption to the family, school, and occupational lives of the IDD community, little is known about the long-term impacts of COVID-19. To address this question, 249 caregivers were surveyed via an online questionnaire, between April and August of 2022 (more than 2 years into the pandemic) about potential impacts of the COVID-19 pandemic on their child's access to health- and school-based therapeutic services, caregiver mental health, and family life. The majority of caregivers reported disruptions in access to and quality of school-based therapeutic services for their child as well as a reduction in educational accommodations in the 2021–2022 academic year. Nearly half of caregivers reported feeling anxious and almost a quarter reported feeling depressed for the majority of their days. More than half of respondents reported decreased social support, and one-fifth reported employment disruptions and decreased access to food. These findings suggest that families of children with IDD are still experiencing ongoing negative impacts of the pandemic, emphasizing the critical need for continued support in the wake of the initial and more obvious disruptions caused by the COVID-19 outbreak.

## 1. Introduction

Intellectual disability is characterized by difficulties with intellectual functioning (e.g., learning and problem solving) and adaptive functioning (e.g., social skills and activities of daily living), while the term “developmental disability” encompasses a broader category that includes both intellectual and physical challenges (1). Intellectual/developmental disability, or IDD, often describes situations in which both intellectual and other developmental disabilities are present. Examples of IDDs include Down syndrome, autism spectrum disorder, and cerebral palsy (1). These conditions are typically present at birth or begin during childhood, and impact individuals in a variety of ways, including how they interact with their family and community (1). Individuals with IDD often have co-occurring medical conditions such as hypertension, heart disease, respiratory disease, and diabetes. The medical complexity associated with IDD places them at greater risk than their neurotypical peers for worse COVID-19 outcomes (2).

Caring for a child with IDD also impacts family life in a variety of ways. In addition to medical concerns, children with IDD often have more behavior problems than their typically developing peers (3, 4), and such behavior problems have been associated with parental stress (5). Caregiver financial burden and employment concerns have also been associated with the severity of a child's disability or health condition (6, 7). The additive impact of medical, behavioral, and financial concerns related to IDD may contribute to caregiver mental health challenges. In a meta-analysis investigating the relationship between caring for a child with IDD and mental health concerns, 90%–95% of studies found a positive association between caring for a child with IDD and parental depression and/or anxiety (8).

Since 2020, the World Health Organization (WHO) has reported globally that over six million people have succumbed to complications associated with the SARS-CoV-2 virus (9). Certain populations, such as the elderly and racially minoritized groups, have been disproportionately affected by the COVID-19 pandemic. Individuals with IDDs are one such population, as they are more likely to contract the virus, become hospitalized, and have a higher case-fatality rate once infected, when compared to the general population (2, 10).

Children with IDD require a host of clinical and medical services to meet their healthcare needs. A large proportion of these services are delivered in their educational setting. The Individuals with Disabilities Education Act (IDEA) ensures that eligible children with disabilities have access to a free public education through the provision of special education and related services through an individualized education program (11). However, the COVID-19 pandemic resulted in a disruption of the delivery of special education and related services, as well as the provisions of necessary accommodations, modifications, and programmatic supports (12).

Several studies surveying caregivers of children with IDD assessed changes in access to educational and therapeutic services due to the COVID-19 pandemic. A Family Strengths Survey disseminated in Western Pennsylvania indicated that 76% of families of children with disabilities had reduced access to early intervention services and 81% had reduced access to school-based therapies due to COVID-19 school closures (13). In a different survey examining special education services, 60% of caregivers reported that following school closures, their children received fewer special education hours and a significant decrease in the number of related service hours compared to what they were supposed to receive, as indicated by their individualized education program (14). Many caregivers reported a complete loss of services, with 74% of caregivers in one survey reporting that their child lost access to at least one therapy or education service (15), and 42% of caregivers from another survey reporting that their child lost access to all therapy services at some point during the first few months of the pandemic (16).

Studies have also shown that caregivers have concerns about the quality of services their child received during the pandemic. When asked about overall satisfaction with their child's therapeutic services during the pandemic, Murphy et al. found that 44% of caregivers completing an online survey reported low satisfaction with services, while only 21% reported high satisfaction (17). In another survey disseminated online across the U.S., Allison & Levac found over 40% of caregivers attributed declines in their child's motor, behavioral, social, and communication skills to these service changes (16).

Given the substantial impact of COVID-19 on family life and the burden that already exists for caregivers of children with IDD, it is not surprising that the pandemic has resulted in substantial caregiver distress. Chafouleas and Iovino found that caregivers of children with disabilities reported significantly higher levels of depression, anxiety, and stress during the pandemic (18). In another, larger survey, Kalb et al. reported caregivers of children with autism spectrum disorder reported higher levels of overall psychological distress, notably feelings of panic, during the COVID-19 pandemic, when compared to a general sample of caregivers in the U.S. (19). There are numerous reasons why the pandemic has had a specific effect on these caregivers. For instance, loss of childcare supports, income/employment (to support the child's healthcare needs), and increased concerns about infection may have acute impacts on these caregivers.

Although a number of studies have been conducted on the impact of COVID-19 on children with IDD and their families, most of these studies took place at the beginning of the pandemic in 2020, when stay-at-home orders were still in place. Less is known about more recent impacts of the pandemic, especially as schools and healthcare facilities began to reopen for in-person operations. The primary goal of this study was to address this gap by understanding the continued impact of the COVID-19 pandemic on children with IDD and their families in terms of: (1) children's health and school-based services, (2) caregiver mental health, and (3) family life. The current study was conducted as part of the Rapid Acceleration of Diagnostics-Underserved Populations (RADx-UP) program, funded by the National Institutes of Health. The RADx-UP program aims to ensure that all Americans, particularly those most impacted by the pandemic, have access to COVID-19 testing (20). RADx-UP supports over 100 community-engaged research projects, including the current study.

## 2. Methods

Data for this cross-sectional study were gathered via an online survey between April and August 2022. The survey was administered via Qualtrics Survey Platform (21) and consisted of an anonymous questionnaire developed using items from an existing survey as well as original items developed for this survey. Surveys were disseminated to email listservs of five disability-related nonprofit organizations. Two of these organizations were nationally based, while three were statewide parent advocacy groups in Maryland and Missouri. As an incentive for completion of the questionnaire, a $50.00 USD donation was offered to the participant's choice for one of the five aforementioned organizations. The only inclusion criterion for the survey was that the respondent must have a child, aged 4–22, with an intellectual or developmental disability. Only one survey response was allowed per respondent (as determined by the Internet Protocol address). All participants were informed, in the opening of the survey, of the ethics surrounding the purpose, procedures, risks, benefits, voluntariness, and payment involved in this study. Participants acknowledged these disclosures by progressing through the survey. The language used for the ethical disclosure, and this study as a whole, was approved by the local Institutional Review Board (IRB).

### 2.1. Measures

#### 2.1.1. Electronic questionnaire

An electronic questionnaire, which took about 25 min to complete, queried caregivers of children with IDD about demographic information, pandemic-related impacts on services, schooling, caregiver mental health, and family life. Most items in this questionnaire were custom developed. Survey items are available upon request to the corresponding author.

Data were gathered between April and August of 2022, and the reporting period for all items was the last three months. At the beginning of the survey, caregivers were asked to indicate how many of their children with IDD were enrolled in a kindergarten through 12th grade school program (excluding homeschool). If a caregiver noted that they had five or more children with IDD enrolled in school, they were asked to share the ages of their five youngest children. For caregivers with more than one child with a disability enrolled in school, one of their children was randomly selected by the survey algorithm as the focus for the rest of the questionnaire. This algorithm was developed to avoid confusion about which child the survey was asking about if the caregiver was raising multiple children with disabilities.

##### 2.1.1.1. Demographics

Survey demographic variables about the caregiver/informant included age, gender, race, ethnicity, relationship to child, number of children in the household, primary language, current state of residence, region, geographic setting, marital status, highest level of education, and employment status. Demographic variables for the randomly selected child with IDD included age (in years), gender, race, highest level of verbal communication, whether or not the child uses alternate forms of communication, type of school, educational accommodations or services, and diagnoses. See Table 1, Supplementary Tables S1, S2 for item categories.

**Table 1:** Participant characteristics.

Characteristic	*n* (%)
Caregiver age	
Under 30	3 (1.3%)
30–39	47 (19.7%)
40–49	112 (47.1%)
50–59	59 (24.8%)
60–69	15 (6.3%)
70 and older	2 (0.8%)
Highest level of education	
High School or less	9 (3.6%)
Trade School/Associates degree	25 (10.0%)
Some college (no degree)	28 (11.2%)
Bachelor's degree	74 (29.7%)
Master's degree	82 (32.9%)
Doctorate degree	27 (10.8%)
Do not wish to disclose	4 (1.6%)
Employment	
Working full-time (32 h or more per week)	137 (55.0%)
Homemaker or stay-at-home caregiver	43 (17.3%)
Working part-time (less than 32 h per week)	43 (17.3%)
Multi select	14 (5.6%)
Full-time student	3 (1.2%)
Retired	3 (1.2%)
Unemployed and looking for work	2 (0.8%)
Furloughed	1 (0.4%)
On disability	1 (0.4%)
Do not wish to disclose	2 (0.8%)
Number of children in the household	
1	86 (34.5%)
2	105 (42.2%)
3	47 (18.9%)
4	8 (3.2%)
5 or more	3 (1.2%)
Child age	
<=5	18 (7.2%)
6–12	107 (43.0%)
13–18	105 (42.2%)
>18	19 (7.6%)
Child diagnosis	
Autism spectrum disorder	135 (54.2%)
ADHD	124 (49.8%)
Speech or language impairment	91 (36.5%)
Anxiety disorder	77 (30.9%)
Intellectual disability	75 (30.1%)
Other	175 (70.3%)

##### 2.1.1.2. COVID-19 impact on child's health- and school-based services

Five 5-point Likert-scale questions were used to assess the impact of the COVID-19 pandemic on the child's progress towards reaching individualized education program (IEP)/504 plan goals, as well as their access to and quality of school-based therapeutic services and accommodations. Response options for the items included: disrupted a little, disrupted a lot, no change, improved a little, or improved a lot. In addition, three binary choice (yes/no) questions asked participants if their child missed or had delayed regular health or dental visits, specialty or referral appointments (e.g., behavior therapy, mental health services, occupational therapy), or non-COVID-19-related immunizations or vaccines (e.g., Hepatitis A, Varicella) due to reasons related to the COVID-19 pandemic.

##### 2.1.1.3. COVID-19 impact on caregiver mental health

Two 4-point Likert-scale questions were asked to assess caregiver mental wellbeing. These items were taken from the Patient Health Questionnaire-4 (22). The items assess anxiety and depression, with the response options reflecting days in the last week they have experienced symptoms (not at all, several days, more than half the days, nearly every day).

##### 2.1.1.4. COVID-19 impact on family life

A single, multi-select question assessed the impact the COVID-19 pandemic on family life (“How has the COVID-19 pandemic affected you and your household? Check all that apply.”). Domains included childcare challenges, financial impacts, employment disruptions, and changes in work settings. In addition, caregivers were asked about their family's current access to medical care (including dental), mental health care, extended family and non-family social supports, and food. Response options for these five questions were decreased a lot, decreased somewhat, no change, increased a lot, and increased somewhat.

### 2.2. Data analysis

The goal of the study was descriptive in nature. As such, descriptive statistics (means, proportions, and percentages) and graphs were used to describe distributions of survey items. All data analytic procedures took place in the R software program (23).

## 3. Results

### 3.1. Participant characteristics

Two hundred forty-nine caregivers of children with intellectual/developmental disabilities completed the questionnaire (Table 1 and Supplementary Table S1). Of those who began the survey and passed the eligibility screening (i.e., had a child with IDD), the rate of completion was 77.5%. The majority of the caregivers were female (90%; 82% biological mothers), White (75%), married (77%), spoke English (93%), living in suburban settings (63%), and had a bachelor's degree or higher (73%). Participants were from the Northeast (61%), West (13%), Midwest (12%), Mountain Region (9%), and Southeast (6%). The state with the most participants was Maryland (44.9%). See Supplementary Table S1 for complete list of states respondents were from.

The majority of the children were white (66%), verbal (88%), attended public school (80%), and had an IEP, 504 plan, or IDEA accommodation (96%; see Table 1 and Supplementary Table S2). Eighty-six percent of children had more than one disability diagnosis, and children had an average of three disabilities. The most common diagnosis was autism spectrum disorder (54%); the five most common diagnoses are listed in Table 1, and the full list of child diagnoses are available in Supplementary Table S2. See Supplementary Table S2 for full child demographics.

### 3.2. Impact on child's health- and school-based services

When asked about impacts on children's school-based services and healthcare services, the majority of caregivers reported disruptions in access and quality of school-based therapeutic services (access: 76%; quality: 75%) and IDEA accommodations (access: 70%; quality: 75%) (Table 2). Progress reaching IEP or 504 plan goals was disrupted for 81% of the sample. Caregivers reported a reduction in medical (43%) and mental health services (37%). Over one-third (37%) reported a delayed or missed regular healthcare or dental visit and 42% reported a delayed or missed specialty or referral appointment.

**Table 2:** Impact on Child's health- and school-based services.

Service	*n* (%)
Access—School-based therapeutic services	
Disrupted a little	60 (30.8%)
Disrupted a lot	88 (45.1%)
No change	41 (21.0%)
Improved a little	2 (1.0%)
Improved a lot	4 (2.1%)
Quality—School-based therapeutic services	
Disrupted a little	52 (26.7%)
Disrupted a lot	94 (48.2%)
No change	44 (22.6%)
Improved a little	3 (1.5%)
Improved a lot	2 (1.0%)
Access—Accommodations in the Classroom	
Disrupted a little	26 (29.9%)
Disrupted a lot	35 (40.2%)
No change	25 (28.7%)
Improved a little	0 (0.0%)
Improved a lot	1 (1.1%)
Quality—Accommodations in the classroom	
Disrupted a little	29 (33.3%)
Disrupted a lot	36 (41.4%)
No change	21 (24.1%)
Improved a little	0 (0.0%)
Improved a lot	1 (1.1%)
Progress—Reaching IEP or 504 plan goals	
Disrupted a little	67 (31.9%)
Disrupted a lot	103 (49.0%)
No change	28 (13.3%)
Improved a little	5 (2.4%)
Improved a lot	7 (3.3%)
Regular healthcare or dental visits	
No	154 (63.4%)
Yes	89 (36.6%)
Specialty appointments or referral visits	
No	139 (58.2%)
Yes	100 (41.8%)
Non-COVID-related immunizations/vaccines (e.g., Hep A, Varicella)	
No	195 (85.9%)
Yes	32 (14.1%)

### 3.3. Impact on caregiver mental health

With respect to the pandemic's impact on caregivers' feelings of anxiety and depression, and how often these feelings occurred, nearly half (45%) of caregivers reported being anxious and over one-fifth (22%) reported being depressed for more than half their days or nearly every day (Table 3).

**Table 3:** Impact on caregiver mental health.

Symptom	*n* (%)
Nervous, anxious, or on edge	
Not at all	32 (12.9%)
Several days	105 (42.2%)
More than half the days	72 (28.9%)
Nearly every day	40 (16.1%)
Down, depressed, or hopeless	
Not at all	82 (32.9%)
Several days	113 (45.4%)
More than half the days	42 (16.9%)
Nearly every day	12 (4.8%)

### 3.4. Impact on family life

Twenty-six percent of participants reported a childcare disruption, 23% reported an adverse financial impact, 41% reported an employment disruption, and 43% reported a disruption in work setting (Table 4). Over half (59%) reported reduced social support and nearly a fifth (18%) reported reduced food access.

**Table 4:** Impact on family life.

Type of Impact	*n* (%)
**Child Care**	**64** (**25.7%)**
Had difficultly arranging for childcare	60 (24.1%)
Incurred increased cost for childcare expenses	25 (10.0%)
**Financial Impact**	**56** (**22.5%)**
Incurred increased cost for childcare expenses	25 (10.0%)
Had difficulty paying bills	34 (13.7%)
Had serious financial problems	17 (6.8%)
**Employment Disruption**	**103** (**41.4%)**
Worked more hours than usual	55 (22.1%)
Worked reduced hours	23 (9.2%)
Started a new job	18 (7.2%)
Income or pay reduced	16 (6.4%)
Was not able to work	11 (4.4%)
Not paid at all	5 (2.0%)
Laid off	3 (1.2%)
Furloughed with full or partial pay	1 (0.4%)
Furloughed without pay	1 (0.4%)
**Work Setting**	**108** (**43.4%)**
Worked remotely/from home more than you usually do	77 (30.9%)
Started working remotely/from home	39 (15.7%)
Access to medical health care (including dental):	
Decreased a lot	22 (9.1%)
Decreased somewhat	83 (34.3%)
No Change	120 (49.6%)
Increased a lot	5 (2.1%)
Increased somewhat	12 (5.0%)
Access to mental health care or treatment:	
Decreased a lot	30 (13.7%)
Decreased somewhat	50 (22.8%)
No Change	118 (53.9%)
Increased a lot	8 (3.7%)
Increased somewhat	13 (5.9%)
Access to extended family and non-family social supports:	
Decreased a lot	58 (24.0%)
Decreased somewhat	84 (34.7%)
No Change	77 (31.8%)
Increased a lot	12 (5.0%)
Increased somewhat	11 (4.5%)
Access to food:	
Decreased a lot	8 (3.3%)
Decreased somewhat	36 (14.9%)
No Change	182 (75.5%)
Increased a lot	4 (1.7%)
Increased somewhat	11 (4.6%)

Bolded numbers under Type of Impact represent aggregates of subcategories below. Subcategories were not mutually exclusive.

## 4. Discussion

COVID-19 has had a significant impact on daily life for children with IDD and their families. However, less is known about the long reach of COVID-19 on families, particularly children with IDD and their caregivers. The current study provides updated data on how the pandemic has continued, two and a half years later, to impact school-based therapeutic services, healthcare services, caregiver mental health, and general family life such as employment and access to social support. Overall, our data suggest that families are still being negatively impacted by the COVID-19 pandemic across multiple domains of life.

Prior research has shown that the COVID-19 pandemic caused major disruptions in educational and therapeutic service delivery, and in some cases has resulted in a loss of services entirely (15). In the current study, three-quarters of caregiver respondents reported that in early to mid-2022, their child's access to school-based therapies and the quality of these services was significantly disrupted or diminished due to the COVID-19 pandemic. More than 8 in 10 caregivers also reported interruptions in their child's progress toward IEP or 504 plan goals. These findings are consistent with literature earlier in the pandemic where caregivers reported disruptions in school-based services and subsequent declines in their child's functioning due to the COVID-19 pandemic (13–17).

Even with a return to in-person education, our data indicate that disruptions continued to occur among children with IDD. This warrants further investigation into potential causes. For instance, factors such as sickness or COVID-19 isolation protocols may result in staff or student absence, and thus contribute to continued disruptions in-person school-based service delivery. Workforce shortages are also more common post-pandemic, including in schools. Additionally, the pandemic has dramatically shifted the ways in which services are delivered, such that many services are now offered virtually. It is possible that caregiver perceptions of service quality are related to the virtual format where technical difficulties may arise, and where parental involvement is crucial, compared to in-person service delivery from only a teacher or provider. Studies have begun to examine differences in service quality between in-person and virtual formats. For instance, caregivers in one survey indicated that barriers to distance learning were related to inconvenient timing of sessions, session attendance being optional, and the child not wanting to participate (24). Given that educational and other school-based services lead to better developmental outcomes for children with IDD, it is critical that we continue to investigate pandemic-induced disruptions.

The current study also revealed healthcare service impacts for children with IDD and their families. Thirty-seven percent of children missed or delayed a regular healthcare or dental visit, and four out of ten missed or delayed a specialty appointment or referral visit. These findings are striking compared to research conducted earlier in the pandemic on missed appointments in general pediatric populations, where between a quarter and a third of children experienced delayed or missed appointments for COVID-19 related reasons (25, 26). Our findings demonstrate that the effects of COVID-19 on missed healthcare appointments could be greater for children with IDD, even years since the start of the pandemic. Since children with IDD often have co-occurring conditions and require more frequent follow-up visits as well as care management, it is essential supports are put in place to ensure they are getting the care they need. Considering that many early pandemic obstacles (e.g., office closures) are less common today, future research is needed to replicate this finding and determine why the pandemic continues to disrupt families' access to regular, specialty, dental, or referral care.

Caregivers in our study also reported negative impacts to their own mental health, consistent with research conducted prior to, and earlier in the pandemic (8, 18, 19). Nearly half (45%) of caregivers indicated that they experienced significant anxiety, whereas about one in five (22%) reported significant symptoms of depression, during early to mid-2022. These mental health challenges may be related to financial, social, or other household strains that have emerged during, and continued throughout the pandemic, such as difficulty paying bills or lack of social support. In the current study, 23% of caregivers reported COVID-19 related financial impacts, and 59% of caregivers reported decreased social support. Although we did not assess relationships between survey responses, it is likely that these financial and social burdens are associated with caregivers' mental health challenges. Additionally, caregivers struggling with their mental health may not have been able to receive the help they needed, as 37% reported a decrease in mental health care or treatment. Future research should assess what factors are contributing to anxiety and depression symptoms in this population, as well as interventions that can help with caregiver burnout and mental health status.

Little research has been published describing COVID-19-related impacts several years since the start of the pandemic. However, one parent-report survey investigating a general population of children in Switzerland found that 12.7% of children were severely impacted by the pandemic two years later across broad domains of child and family functioning (27). These figures are far lower than the proportions found in our study on children with IDD, such as the 81% who faced disruptions in progress reaching their IEP or 504 plan goals, or the 42% who missed specialty appointments or referral appointments. While we do not have data on our sample prior to COVID-19 to compare pre-pandemic trends, it is possible that existing challenges associated with raising a child with IDD were exacerbated due to the pandemic.

A major strength of this study is that it fills an important gap in the literature by addressing the long-term effects of the pandemic on a vulnerable population. Although stay-at-home orders have long been lifted, it is important to understand how families of children with IDD are still being impacted by the pandemic, so that we can develop effective strategies to support their needs. Another strength of this study is that we were able to represent a relatively wide range of children in terms of ages and diagnoses. With large and almost equivalent samples of younger school-aged children (43% ages 6–12) and older school-aged children (42% ages 13–18), as well as small samples of children under five (7%) and over 18 (8%), we were able to represent children at different developmental levels, and thus demonstrate that our findings are not limited to a certain age range or school level. Our sample also consisted of children diagnosed with a variety of different IDDs, and thus we were able to represent a variety of disability-related experiences and perspectives.

This study also has its limitations. Most notably, our findings may not generalize to all children with IDD and their families. Although the survey was distributed to multiple, disability-related organizations, sampling bias may have been present. Members of these organizations, and those who chose to complete the survey, may share certain characteristics that are not representative of all families of children with IDD. The majority of our respondents were female (90%), White (75%), and highly educated (73% had a bachelor's degree or higher), and therefore do not represent all caregivers of children with IDD. It is possible that marginalized groups such as people of color or those with lower levels of educational attainment may face even greater challenges due to the COVID-19 pandemic. Another limitation is the lack of data available on the children's level of support needs, which may have been associated with the outcomes described in the current study. For example, it is possible that children with greater support needs (i.e., children who receive more services) may have faced additional disruptions in service access and quality than children with fewer support needs. In turn, children with greater support needs may have had more difficulty progressing towards their academic or behavioral goals. Similarly, families' pandemic-related experiences may have varied based on their child's condition. To capture a variety of perspectives, we included children with a wide range of IDDs; however, we did not assess potential differences in COVID-19-related impacts between these diagnostic groups. Additionally, our study did not have a comparison group including caregivers of typically developing children, so it is unclear whether these impacts are exclusive to families of children with IDD, or if they are prevalent amongst all families. Lastly, since the majority of survey questions were novel, we cannot ensure their validity or reliability.

In summary, the present study queried caregivers of children with IDD on recent impacts of the COVID-19 pandemic on school-based therapeutic and healthcare services, caregiver mental health, and family life. Notably, these data are reflective of recent months, indicating ongoing and long-lasting impacts. While much of the existing literature focuses on the broadly experienced impacts of the early pandemic, it is essential to understand how and why families of children with IDD continue to experience negative impacts to provide data-informed support to this already-vulnerable population. Although the majority of schools and healthcare facilities are open again, there are still a number of pandemic-related influences that continue to shape the delivery of care, special education, related services, and day-to-day life as we know it. Ongoing investigation of the delivery and quality of school-based therapeutic services, caregiver mental health, and family life disruptions will provide the data needed to support children with IDD and their families as they continue to navigate the COVID-19 pandemic.

## Data Availability

The datasets presented in this article are not readily available because Consent was not obtained for sharing of information. Requests to access the datasets should be directed to kalb@kennedykrieger.org.
