# Supplementary material for: Caregiver perspectives on the continued impact of the COVID-19 pandemic on children with intellectual/developmental disabilities
Source: Front Pediatr. 2023 Aug 7;11:1196275. doi: 10.3389/fped.2023.1196275 (PMC10440736; doi:10.3389/fped.2023.1196275)
Supplement: Supplementary file 1 [file Table1.docx]

**Table S1. Caregiver Characteristics (N=249)**

| Characteristic | n (%) |
| --- | --- |
| Age |  |
| Under 21 | 3 (1.3%) |
| 30-39 | 47 (19.7%) |
| 40-49 | 112 (47.1%) |
| 50-59 | 59 (24.8%) |
| 60-69 | 15 (6.3%) |
| 70 and older | 2 (0.8%) |
| Gender |  |
| Female | 223 (89.6%) |
| Male | 16 (6.4%) |
| Other / Do not wish to disclose | 10 (4.0%) |
| Race |  |
| White | 187 (75.1%) |
| Black or African American | 23 (9.2%) |
| Multi-Racial | 13 (5.2%) |
| Asian | 7 (2.8%) |
| Other | 2 (0.8%) |
| American Indian/Alaskan Native | 1 (0.4%) |
| Do not wish to disclose | 16 (6.4%) |
| Ethnicity |  |
| Non-Hispanic or Non-Latino/Latina/Latinx | 204 (81.9%) |
| Hispanic or Latino/Latina/Latinx | 20 (8.0%) |
| Do not wish to disclose | 25 (10.0%) |
| Relationship to child |  |
| Biological Mother | 202 (81.5%) |
| Adoptive Mother | 20 (8.1%) |
| Biological Father | 9 (3.6%) |
| Grandmother | 6 (2.4%) |
| Adoptive Father | 3 (1.2%) |
| Legally Authorized Representative | 3 (1.2%) |
| Stepmother | 3 (1.2%) |
| Other | 2 (0.8%) |
| Number of children in the household |  |
| 1 | 86 (34.5%) |
| 2 | 105 (42.2%) |
| 3 | 47 (18.9%) |
| 4 | 8 (3.2%) |
| 5 or more | 3 (1.2%) |
| Language |  |
| English | 231 (92.8%) |
| English / Spanish | 10 (4.0%) |
| English / Other | 8 (3.2%) |
| State of Residence: |  |
| Maryland | 111 (44.9%) |
| California | 20 (8.1%) |
| Missouri | 16 (6.5%) |
| Texas | 13 (5.3%) |
| Massachusetts | 8 (3.2%) |
| Pennsylvania | 6 (2.4%) |
| Washington | 6 (2.4%) |
| Other | 67 (27.1%) |
| Region |  |
| Northeast | 150 (60.7%) |
| West | 31 (12.6%) |
| Midwest | 30 (12.1%) |
| Mountain Region | 22 (8.9%) |
| Southeast | 14 (5.7%) |
| Geographic setting (self-described) |  |
| Suburban | 157 (63.3%) |
| Rural | 42 (16.9%) |
| Urban | 42 (16.9%) |
| Unsure | 4 (1.6%) |
| Do not wish to disclose | 3 (1.2%) |
| Marital status |  |
| Married/Living together, not married | 189 (76.5%) |
| Single | 28 (11.3%) |
| Divorced | 17 (6.9%) |
| Separated | 2 (0.8%) |
| Widowed | 1 (0.4%) |
| Do not wish to disclose | 10 (4.0%) |
| Highest level of education |  |
| High School or less | 9 (3.6%) |
| Trade School/Associates degree | 25 (10.0%) |
| Some college (no degree) | 28 (11.2%) |
| Bachelor's degree | 74 (29.7%) |
| Master's degree | 82 (32.9%) |
| Doctorate degree | 27 (10.8%) |
| Do not wish to disclose | 4 (1.6%) |
| Employment |  |
| Working full-time (32 hours or more per week) | 137 (55.0%) |
| Homemaker or stay-at-home caregiver | 43 (17.3%) |
| Working part-time (Less than 32 hours per week) | 43 (17.3%) |
| Multi Select | 14 (5.6%) |
| Full-time student | 3 (1.2%) |
| Retired | 3 (1.2%) |
| Unemployed and looking for work | 2 (0.8%) |
| Furloughed | 1 (0.4%) |
| On disability | 1 (0.4%) |
| Do not wish to disclose | 2 (0.8%) |
