# Supplementary material for: Caregiver perspectives on the continued impact of the COVID-19 pandemic on children with intellectual/developmental disabilities
Source: Front Pediatr. 2023 Aug 7;11:1196275. doi: 10.3389/fped.2023.1196275 (PMC10440736; doi:10.3389/fped.2023.1196275)
Supplement: Supplementary file 2 [file Table2.docx]

**Table S2. Child Characteristics (N=249)**

| Characteristic | n (%) |
| --- | --- |
| Age |  |
| <=5 | 18 (7.2%) |
| 6-12 | 107 (43.0%) |
| 13-18 | 105 (42.2%) |
| >18 | 19 (7.6%) |
| Gender |  |
| Male | 170 (68.3%) |
| Female | 68 (27.3%) |
| Other / Do not wish to disclose | 11 (4.4%) |
| Race |  |
| White | 163 (65.5%) |
| Multi-Racial | 30 (12.0%) |
| Black or African American | 26 (10.4%) |
| Asian | 8 (3.2%) |
| American Indian/Alaskan Native | 3 (1.2%) |
| Other | 1 (0.4%) |
| Do not wish to disclose | 18 (7.2%) |
| Highest level of verbal communication: |  |
| Gestures | 20 (8.0%) |
| Single Words | 8 (3.2%) |
| Short Phrases | 37 (14.9%) |
| Full Sentences | 173 (69.5%) |
| Alternate Communication Only | 11 (4.4%) |
| Child uses alternate forms of communication (ASL, AAC, PECS): |  |
| No | 199 (79.9%) |
| Yes | 50 (20.1%) |
| Type of school/preschool child currently attends |  |
| Public school | 194 (79.8%) |
| Private school | 32 (13.2%) |
| Homeschool or co-op program | 9 (3.7%) |
| Charter school | 5 (2.1%) |
| Unsure | 3 (1.2%) |
| Receipt of the following services: |  |
| IEP or 504 plan only | 132 (57.1%) |
| IEP or 504 plan, and IDEA accommodations | 85 (36.8%) |
| IDEA accommodations only | 3 (1.3%) |
| None | 9 (3.9%) |
| Unsure | 2 (0.9%) |
| Diagnoses |  |
| Autism spectrum disorder | 135 (54.2%) |
| ADHD | 124 (49.8%) |
| Speech or language impairment | 91 (36.5%) |
| Anxiety disorder | 77 (30.9%) |
| Intellectual disability | 75 (30.1%) |
| Developmental delay | 68 (27.3%) |
| Specific learning disabilities | 62 (24.9%) |
| Genetic disorder | 34 (13.7%) |
| Epilepsy or seizure disorder | 33 (13.3%) |
| Other (please note): | 27 (10.8%) |
| Behavioral or conduct disorder | 24 (9.6%) |
| Visual impairment/blindness | 22 (8.8%) |
| Orthopedic impairments | 21 (8.4%) |
| Cerebral palsy | 17 (6.8%) |
| Down syndrome | 17 (6.8%) |
| Emotional disturbance | 13 (5.2%) |
| Hearing impairment/deafness | 13 (5.2%) |

Note: Characteristics refer to those of the selected child (if more than one child per household). Child diagnoses included only if more than 5% of the sample endorsed them.
